# The Generalized Sidelobe Canceller Based on Quaternion Widely Linear Processing

**DOI:** 10.1155/2014/942923

**Published:** 2014-04-29

**Authors:** Jian-wu Tao, Wen-xiu Chang

**Affiliations:** ^1^Department of Flight Vehicle Control, Aviation University, Changchun, Jilin 130022, China; ^2^College of Communication Engineering, Jilin University, Changchun, Jilin 130025, China

## Abstract

We investigate the problem of quaternion beamforming based on widely linear processing. First, a quaternion model of linear symmetric array with two-component electromagnetic (EM) vector sensors is presented. Based on array's quaternion model, we propose the general expression of a quaternion *semiwidely* linear (QSWL) beamformer. Unlike the complex widely linear beamformer, the QSWL beamformer is based on the simultaneous operation on the quaternion vector, which is composed of two jointly proper complex vectors, and its involution counterpart. Second, we propose a useful implementation of QSWL beamformer, that is, QSWL generalized sidelobe canceller (GSC), and derive the simple expressions of the weight vectors. The QSWL GSC consists of two-stage beamformers. By designing the weight vectors of two-stage beamformers, the interference is completely canceled in the output of QSWL GSC and the desired signal is not distorted. We derive the array's gain expression and analyze the performance of the QSWL GSC in the presence of one type of interference. The advantage of QSWL GSC is that the main beam can always point to the desired signal's direction and the robustness to DOA mismatch is improved. Finally, simulations are used to verify the performance of the proposed QSWL GSC.

## 1. Introduction

As an important tool of multidimensional signal processing, the quaternion algebra has been applied to parameter estimation of 2D harmonic signals [1], DOA estimation of polarized signals [2–4], image processing [5], space-time-polarization block codes [6], Kalman filter [7], adaptive filter [8, 9], independent component analysis (ICA) algorithm [10], widely linear modeling and filtering [11–15], nonlinear adaptive filtering [16], and blind source separation [17]. In these applications, the interest in quaternion widely linear processing has recently increased due to the use of the full second-order statistical information in the quaternion domain. In [13], a widely linear quaternion least mean square (WL-QLMS) algorithm was presented to improve the accuracy in adaptive filtering of both second-order circular (Q-proper) and second-order noncircular (Q-improper) quaternion signals. In [14], a class of variable step-size algorithms were introduced into the WL-QLMS, so as to enhance the WL-QLMS tracking ability and sensibility to dynamically changing environments. In [15], a reduced-complexity WL-QLMS algorithm was developed to reduce computational cost and speed convergence of the WL-QLMS. In [16], a nonlinear quaternion adaptive filter and its widely linear version were proposed based on locally analytic nonlinear activation function. In [17], a quaternion widely linear predictor was applied to the blind source separation to extract both Q-proper and Q-improper sources. All the quaternion widely linear algorithms employ the quaternion widely linear model and the associated augmented quaternion statistics, which includes the information in both the standard covariance and the three pseudocovariances, so that their performance was enhanced. Motivated by the benefits of signal processing in quaternion domain, the quaternion beamformers [18–20] were recently developed. In [18], a quaternion minimum mean square error algorithm was proposed and applied to the beamforming of an airborne trimmed vector-sensor array. In [19], a quaternion-capon beamformer using a crossed dipole array was proposed to improve the robustness of Capon beamformer. In [20], an interference and noise cancellation algorithm of quaternion MVDR beamformer was proposed to cancel the uncorrelated interference. Because the only information in the standard covariance of quaternion signals is used, the performance of these beamformers is not obviously enhanced. To make full use of the information in both the standard covariance and the three pseudocovariances of quaternion signals, the quaternion widely linear model may be introduced in the quaternion beamformer. Unfortunately, the quaternion beamformer based on widely linear model has received a little attention.

In this paper, we investigate the problem of quaternion beamforming based on widely linear processing. Because the quaternion output vector of array with two-component EM vector sensors is constructed by two jointly proper complex vectors, we present firstly the general expression of a quaternion* semiwidely* linear (QSWL) beamformer in this paper. Unlike the complex widely linear beamformers in [21–23], the QSWL beamformer can handle the proper complex vectors, whereas in this case the complex widely linear beamformer degenerates to conventional linear processing due to the vanishing of pseudocovariance. Thus, the QSWL beamformer can employ more information than the complex widely linear beamformers to improve the performance. Moreover, we propose a useful implementation of QSWL beamformer, that is, QSWL generalized sidelobe canceller (GSC), using a linear symmetric array. The QSWL GSC consists of two-stage beamformers. By designing the weight vectors of two-stage beamformers, the interference is completely canceled in the output of QSWL GSC and the desired signal is not distorted.

## 2. The Quaternion Widely Linear Beamformer for Vector-Sensor Array

### 2.1. Quaternion Model of Vector-Sensor Array

**Figure 1:**
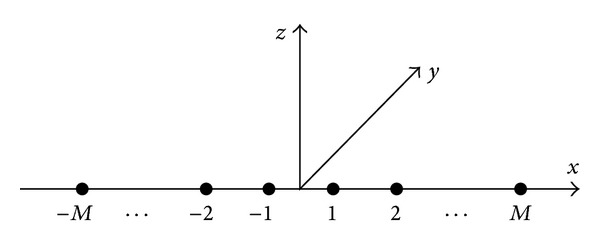
A uniform linear symmetric array.

Consider that a scenario with one narrowband, completely polarized source, which is traveling in an isotropic and homogeneous medium, impinges on a uniform linear symmetric array from direction (*θ*, *ϕ*). This uniform linear symmetric array consists of 2*M* two-component vector sensors, which is depicted in Figure 1, and the spacing between the adjacent two vector sensors is assumed to be half wavelength. The array's reference point is at the array centroid and all vector sensors are indexed by −*M*,…, −1,1,…, *M* from left to right. Two complex series *x*_*m*1_(*n*) and *x*_*m*2_(*n*) are obtained from first and second components of the *m*th two-component vector sensor, respectively. The complex series *x*_*m*1_(*n*) and *x*_*m*2_(*n*) in the planes spanned by {1, *i*}, where *i* denotes a pure unit imaginary, are given by

(1) $$\begin{array}{c} \left[\begin{array}{c} x_{m1}\left(n\right)\\ x_{m2}\left(n\right) \end{array}\right]=\left[\begin{array}{c} a_{1}\left(\theta ,\phi ,\alpha ,\beta \right)\\ a_{2}\left(\theta ,\phi ,\alpha ,\beta \right) \end{array}\right]q_{m}\left(\theta ,\phi \right)s\left(n\right), \end{array}$$

where *θ* and *ϕ* denote the incidence source's elevation angle measured from the positive *z*-axis and azimuth angle measured from the positive *x*-axis, respectively. *α* represents the orientation angle, and *β* signifies the ellipticity angle. *a*_1_(*θ*, *ϕ*, *α*, *β*) and *a*_2_(*θ*, *ϕ*, *α*, *β*) are the responses on first and second components of two-component vector sensor, respectively. *q*_*m*_(*θ*, *ϕ*) is the spatial phase factor describing wavefield propagation along an array, and *q*_*m*_(*θ*, *ϕ*) = *q*_−m_*(*θ*, *ϕ*) due to the symmetric structure of uniform linear array. *s*(*n*) is the complex envelope of the waveform, assumed to be a stationary stochastic process with zero mean and second-order circularity. Thus, *x*_*m*1_(*n*) and *x*_*m*2_(*n*) are also a stationary stochastic process with zero mean and second-order circularity.

Using *x*_*m*1_(*n*) and *x*_−*m*2_(*n*) (*m* = −*M*,…, *M*), the output of the *m*th two-component vector sensor can be written in the quaternion's* Cayley-Dickson* representation as

(2) xm(n)=xm1(n)+jx−m2(n)=qm(θ,ϕ)(a1(θ,ϕ,α,β)+ja2(θ,ϕ,α,β))s(n)=qm(θ,ϕ)P(θ,ϕ,α,β)s(n),

where *j* denotes a pure unit imaginary in a quaternion domain ^1^. *P*(*θ*, *ϕ*, *α*, *β*) = *a*_1_(*θ*, *ϕ*, *α*, *β*) + *ja*_2_(*θ*, *ϕ*, *α*, *β*) is the quaternion-valued response on a two-component vector sensor. This transformation maps the complex signal *x*_*m*1_(*n*) on scalar and *i* imaginary fields of a quaternion, and the complex signal *x*_−*m*2_(*n*) is simultaneously mapped to the *j* and *k* imaginary fields of a quaternion. It is noted that *x*_*m*_(*n*) is *C* ^*i*^-proper ^2^ because two complex series *x*_*m*1_(*n*) and *x*_−*m*2_(*n*) are second-order circularity. When the quaternion-valued additive noise is considered, *x*_*m*_(*n*) can be rewritten as

(3) $$\begin{array}{c} x_{m}\left(n\right)=q_{m}\left(\theta ,\phi \right)P\left(\theta ,\phi ,\alpha ,\beta \right)s\left(n\right)+n_{m}\left(n\right), \end{array}$$

where *n*_*m*_(*n*) = *n*_*m*1_(*n*) + *jn*_−*m*2_(*n*). *n*_*m*1_(*n*) and *n*_−*m*2_(*n*) are, respectively, the complex-valued additive noises at the first component of the *m*th vector sensor and the second component of the −*m*th vector sensor, which are assumed to be zero mean, Gaussian noise with identical covariance *σ*_*n*_^2^. And it is assumed that *n*_*m*_(*n*) and *n*_*k*_(*n*), where *m* ≠ *k*, are uncorrelated.

In order to extend this model to the multisource case, we assume that *K* uncorrelated, completely polarized plane waves impinge on this array. One is the desired signal characterized by its arrival angles (*θ*_*d*_, *ϕ*_*d*_) and polarization parameters (*α*_*d*_, *β*_*d*_); the others are the interference characterized by its arrival angles (*θ*_*u*_, *ϕ*_*u*_) and polarization parameters (*α*_*u*_, *β*_*u*_), where *u* = 2,…, *K*. Thus, the quaternion-valued measurement vector of array can be written as

(4) x(n)=[x−M(n),…,xM(n)]T=q(θd,ϕd)P(θd,ϕd,αd,βd)sd(n) +∑u=2Kq(θu,ϕu)P(θu,ϕu,αu,βu)su(n)+N(n)=vdsd(n)+∑u=2Kvusu(n)+N(n),

where **q**(*θ*, *ϕ*) = [*q*_−*M*_(*θ*, *ϕ*),…, *q*_*M*_(*θ*, *ϕ*)]^*T*^ denotes the spatial phase vector. **v**_*d*_ = **q**_*d*_ *P*_*d*_ and **v**_*u*_ = **q**_*u*_ *P*_*u*_ are the quaternion-valued steering vector associated with the desired signal and the interference, respectively, where **q**(*θ*_*k*_, *ϕ*_*k*_) and *P*(*θ*_*k*_, *ϕ*_*k*_, *α*_*k*_, *β*_*k*_) are denoted by **q**_*k*_ and *P*_*k*_ (*k* = *d*, *u*), respectively. **N**(*n*) = [*n*_−*M*_(*n*),…, *n*_*M*_(*n*)]^*T*^ denotes the quaternion-valued additive noise vector. Because *x*_*m*_(*n*) (*m* = −*M*,…, *M*) is *C* ^*i*^-proper, **x**(*n*) is also *C* ^*i*^-proper vector.

### 2.2. The Quaternion Semiwidely Linear Beamformer

The most general linear processing is* full widely* linear processing, which consists in the simultaneous operation on the quaternion vector and its three involutions. Then, a quaternion widely linear beamformer can be written as

(5) y(n)=W⊲x(n)+G⊲x(i)(n) +H⊲x(j)(n)+F⊲x(k)(n),

where **W**, **G**, **H**, and **F** are the quaternion-valued weight vectors. Superscript (·)^⊲^ denotes the quaternion conjugate and transpose operator. **x** ^(*i*)^(*n*), **x** ^(*j*)^(*n*), and **x** ^(*k*)^(*n*) denote the quaternion involution of **x**(*n*) over a pure unit imaginary *i*, *j*, and *k*, respectively.

The* full widely* linear processing is optimal processing for the Q-improper quaternion vector. Since the quaternion-valued vector **x**(*n*) is *C* ^*i*^-proper vector, the optimal processing reduces to* semiwidely linear* processing ^3^. Because the* semiwidely* linear processing consists only in the simultaneous operation on the quaternion vector and its involution over *i*, the general expression of a quaternion* semiwidely* linear (QSWL) beamformer can be written as

(6) $$\begin{array}{c} y\left(n\right)=W^{⊲}x\left(n\right)+G^{⊲}x^{\left(i\right)}\left(n\right), \end{array}$$

where **x** ^(*i*)^(*n*) is given by [11]

(7) x(i)(n)=−ix(n)i=vd(i)sd(n)+∑u=2Kvu(i)su(n)+N(i)(n).

Moreover, we can write the quaternion-valued output series *y*(*n*) in the following* Cayley-Dickson* representation:

(8) y(n)=([W⊲x(n)]1+[G⊲x(i)(n)]1) +j([W⊲x(n)]2+[G⊲x(i)(n)]2)=y1(n)+jy2(n),

where [*x*]_1_ and [*x*]_2_ denote, respectively, the first and the second complex-valued components of a quaternion *x*; that is, *x* = [*x*]_1_ + *j*[*x*]_2_. Thus, the QSWL beamformer has two complex-valued output series *y*_1_(*n*) and *y*_2_(*n*) in the planes spanned by {1, *i*}, where *y*_1_(*n*) = [**W** ^⊲^ **x**(*n*)]_1_ + [**G** ^⊲^ **x** ^(*i*)^(*n*)]_1_ and *y*_2_(*n*) = [**W** ^⊲^ **x**(*n*)]_2_ + [**G** ^⊲^ **x** ^(*i*)^(*n*)]_2_. Since the conventional “long vector" beamformer has only one complex-valued output series *y*_1_(*n*), the QSWL beamformer can obtain more information than the conventional “long vector” beamformer. The increase of information results in the improvement of QSWL beamformer's performance. In addition, we incorporate the information on both **x**(*n*) and **x** ^(*i*)^(*n*), so that the QSWL beamformer with different characteristics may be obtained by designing two weight vectors **W** and **G** under some different criterions.

## 3. The QSWL Generalized Sidelobe Canceller

**Figure 2:**
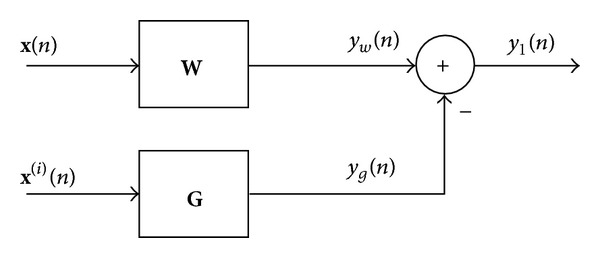
The structure of QSWL GSC.

In this section, a useful implementation of the QSWL beamformer, that is, QSWL generalized sidelobe canceller (GSC), is proposed. The QSWL GSC, which is depicted in Figure 2, consists of two-stage beamformers. In the first-stage beamformer (weight vector is **W**), we attempt to extract a desired signal without any distortion from observed data. To cancel interference, we attempt to estimate interference in second-stage beamformer (weight vector is **G**). By employing the output of the second-stage beamformer to cancel interference in the output of the first-stage beamformer, there is no interference in the output of the QSWL GSC. Compared with the conventional “long vector” beamformer, the advantages of two-stage beamformers are that the main beam can always point to desired signal's direction, even if the separation between the DOAs of the desired signal and interference is less, and the robustness to DOA mismatch is improved.

In the following, we derive the expressions of quaternion-valued weight vectors **W** in the first-stage beamformer and **G** in the second-stage beamformer. It is assumed that two (*K* = 2) uncorrelated, completely polarized plane waves, whose waveform is unknown but whose DOA and polarization may be* priorly* estimated from techniques presented in [2, 4, 25–29], impinge on an array depicted in Figure 1. One plane wave is the desired signal and its complex envelope is denoted by *s*_*d*_(*n*); the other plane wave is the interference and its complex envelope is denoted by *s*_*u*_(*n*). Since *s*_*d*_(*n*) and *s*_*u*_(*n*) are complex series, the output of the QSWL GSC must be complex series. Because the quaternion-valued output has two complex-valued components in the planes spanned by {1, *i*}, we define the first complex-valued output component as the output of the QSWL GSC. Thus, the complex-valued output of the QSWL GSC is written as

(9) $$\begin{array}{c} y_{\text{GSC}}\left(n\right)={\left[y_{Q}\left(n\right)\right]}_{1}=y_{w}\left(n\right)-y_{g}\left(n\right), \end{array}$$

where *y*_*w*_(*n*) is the complex-valued output of the first-stage beamformer; that is, *y*_*w*_(*n*) = [**W** ^⊲^ **x**(*n*)]_1_; *y*_*g*_(*n*) is the complex-valued output of the second-stage beamformer; that is, *y*_*g*_(*n*) = [**G** ^⊲^ **x** ^(*i*)^(*n*)]_1_.

### 3.1. The First-Stage Beamformer

From (4), we have

(10) yw(n)=[W⊲vd]1sd(n)+[W⊲vu]1su(n) +[W⊲N(n)]1.

In the first-stage beamformer, we attempt to minimize the interference-plus-noise energy in *y*_*w*_(*n*), subject to the constraint [**W** ^⊲^ **v**_*d*_]_1_ = 1.

Since the* Cayley-Dickson* representation of **W**, **v**_*d*_, **v**_*u*_, and **N**(*n*) is, respectively, **W** = **W**_1_ + *j* **W**_2_, **v**_*d*_ = **v**_*d*1_ + *j* **v**_*d*2_, **v**_*u*_ = **v**_*u*1_ + *j* **v**_*u*2_, and **N**(*n*) = **N**_1_(*n*) + *j* **N**_2_(*n*), we have

(11) $$\begin{array}{c} {\left[W^{⊲}v_{d}\right]}_{1}=W_{1}^{H}v_{d1}+W_{2}^{H}v_{d2}={\overset{-}{W}}^{H}{\overset{-}{V}}_{d}, \end{array}$$

(12) $$\begin{array}{c} {\left[W^{⊲}v_{u}\right]}_{1}=W_{1}^{H}v_{u1}+W_{2}^{H}v_{u2}={\overset{-}{W}}^{H}{\overset{-}{V}}_{u}, \end{array}$$

(13) $$\begin{array}{c} {\left[W^{⊲}N\left(n\right)\right]}_{1}=W_{1}^{H}N_{1}\left(n\right)+W_{2}^{H}N_{2}\left(n\right)={\overset{-}{W}}^{H}\overset{-}{N}\left(n\right), \end{array}$$

where

(14) $$\begin{array}{c} \overset{-}{W}=\left[\begin{array}{c} W_{1}\\ W_{2} \end{array}\right],\;\;{\overset{-}{V}}_{d}=\left[\begin{array}{c} v_{d1}\\ v_{d2} \end{array}\right],\\ {\overset{-}{V}}_{u}=\left[\begin{array}{c} v_{u1}\\ v_{u2} \end{array}\right],\;\;\overset{-}{N}\left(n\right)=\left[\begin{array}{c} N_{1}\left(n\right)\\ N_{2}\left(n\right) \end{array}\right]. \end{array}$$

Superscript (·)^*H*^ denotes the complex conjugate and transpose operator. Thus, (10) can be rewritten as

(15) $$\begin{array}{c} y_{w}\left(n\right)={\overset{-}{W}}^{H}{\overset{-}{V}}_{d}s_{d}\left(n\right)+{\overset{-}{W}}^{H}{\overset{-}{V}}_{u}s_{u}\left(n\right)+{\overset{-}{W}}^{H}\overset{-}{N}\left(n\right). \end{array}$$

Then, $\overset{-}{W}$ can be derived by solving the following constrained optimization problem:

(16) $$\begin{array}{c} J\left(\overset{-}{W}\right)=\min\left\{{\overset{-}{W}}^{H}R_{in}\overset{-}{W}\right\},\;\text{subject}\;\text{to}\;\;{\overset{-}{W}}^{H}{\overset{-}{V}}_{d}=1, \end{array}$$

where

(17) $$\begin{array}{c} R_{in}=\left[\begin{array}{cc} E\left\{{\left[x_{in}(n)\right]}_{1}{\left[x_{in}(n)\right]}_{1}^{H}\right\} & E\left\{{\left[x_{in}(n)\right]}_{1}{\left[x_{in}(n)\right]}_{2}^{H}\right\}\\ E\left\{{\left[x_{in}(n)\right]}_{2}{\left[x_{in}(n)\right]}_{1}^{H}\right\} & E\left\{{\left[x_{in}(n)\right]}_{2}{\left[x_{in}(n)\right]}_{2}^{H}\right\} \end{array}\right] \end{array}$$

is the covariance matrix and **x**_*in*_(*n*) = **v**_*u*_ *s*_*u*_(*n*) + **N**(*n*) is the measurement vector of array in the absence of the desired signal. The solution of this constrained optimization problem is obtained by using Lagrange multipliers; that is,

(18) $$\begin{array}{c} \overset{-}{W}=\frac{R_{in}^{-1}{\overset{-}{V}}_{d}}{{\overset{-}{V}}_{d}^{H}R_{in}^{-1}{\overset{-}{V}}_{d}}. \end{array}$$

If the interference is uncorrelated with the additive noise, $\overset{-}{W}$ can be written in the simple form (the proof is in Appendix A)

(19) $$\begin{array}{c} \overset{-}{W}=\frac{\varepsilon {\overset{-}{V}}_{d}-{\left[P_{u}^{⊲}P_{d}\right]}_{1}q_{u}^{H}q_{d}{\overset{-}{V}}_{u}}{\mu }, \end{array}$$

where

(20) $$\begin{array}{c} \mu =2M{\left|P_{d}\right|}^{2}\varepsilon -{\left|{\left[P_{u}^{⊲}P_{d}\right]}_{1}\right|}^{2}{\left|q_{u}^{H}q_{d}\right|}^{2},\\ \varepsilon =\xi_{u}^{-1}+2M{\left|P_{u}\right|}^{2}, \end{array}$$

where *ξ*_*u*_ denotes the input interference-to-noise ratio (INR). Moreover, the quaternion-valued optimal weight vector **W** may be given by

(21) $$\begin{array}{c} W=J_{1}\overset{-}{W}+jJ_{2}\overset{-}{W}, \end{array}$$

where **J**_1_ = [**I**_2*M*×2*M*_, 0_2*M*×2*M*_] and **J**_2_ = [0_2*M*×2*M*_, **I**_2*M*×2*M*_] are two selection matrices. It is noted that in some applications, such as Radar, **R**_*in*_ may be estimated in intervals of no transmitted signal. But, **R**_*in*_ is not obtained in other applications, such as Communications. In these applications, we may replace **R**_*in*_ by **R**_*x*_, where

(22) $$\begin{array}{c} R_{x}=\left[\begin{array}{cc} E\left\{{\left[x(n)\right]}_{1}{\left[x(n)\right]}_{1}^{H}\right\} & E\left\{{\left[x(n)\right]}_{1}{\left[x(n)\right]}_{2}^{H}\right\}\\ E\left\{{\left[x(n)\right]}_{2}{\left[x(n)\right]}_{1}^{H}\right\} & E\left\{{\left[x(n)\right]}_{2}{\left[x(n)\right]}_{2}^{H}\right\} \end{array}\right] \end{array}$$

is the covariance matrix and **x**(*n*) = **v**_*d*_ *s*_*d*_(*n*) + **v**_*u*_ *s*_*u*_(*n*) + **N**(*n*) is the measurement vector of array. When the distortionless constraint is perfectly matched with the desired signal, the weight vector **W** is identical in both **R**_*x*_ and **R**_*in*_ [30].

By using the optimal weight vector **W**, the complex output of the first-stage beamformer can be given by

(23) $$\begin{array}{c} y_{w}\left(n\right)=s_{d}\left(n\right)+{\left[W^{⊲}v_{u}\right]}_{1}s_{u}\left(n\right)+{\left[W^{⊲}N\left(n\right)\right]}_{1}. \end{array}$$

### 3.2. The Second-Stage Beamformer

From (7), we have

(24) yg(n)=[G⊲vd(i)]1sd(n)+[G⊲vu(i)]1su(n) +[G⊲N(i)(n)]1.

In the second-stage beamformer, we attempt to minimize the noise energy in *y*_*g*_(*n*), subject to the constraints [**G** ^⊲^ **v**_*d*_^(*i*)^]_1_ = 0 and [**G** ^⊲^ **v**_*u*_^(*i*)^]_1_ = [**W** ^⊲^ **v**_*u*_]_1_. In the following, two schemes are presented to implement this aim.

#### 3.2.1. Scheme  1, That Is, Combined QPMC and MVDR

Let **G** = **w**_*QS*_ **w**_*MV*_, where **w**_*QS*_ is a quaternion-valued diagonal weight matrix and **w**_*MV*_ is a complex weight vector. In this scheme, the first step is to achieve the constraint [**G** ^⊲^ **v**_*d*_^(*i*)^]_1_ = 0 by designing **w**_*QS*_, and this step is referred to quaternion polarization matched cancellation (QPMC); the second step is to minimize the noise energy in *y*_*g*_(*n*) subject to the constraint [**G** ^⊲^ **v**_*u*_^(*i*)^]_1_ = [**W** ^⊲^ **v**_*u*_]_1_ by designing **w**_*MV*_, and this step is referred to MVDR.

Let **w**_*QS*_ = diag⁡{*w*_*QS*_(−*M*),…, *w*_*QS*_(*M*)}; then we have

(25) G⊲vd(i)=wMVHwQS⊲vd(i)=wMVH[wQS∗(−M)q−M(θd,ϕd)Pd(i)⋮wQS∗(M)qM(θd,ϕd)Pd(i)],

where superscript (·)* denotes the quaternion conjugate operator. From (25) and the constraint [**G** ^⊲^ **v**_*d*_^(*i*)^]_1_ = 0, we have the constraint [*w*_QS_*(*m*)*q*_*m*_(*θ*_*d*_, *ϕ*_*d*_)*P*_*d*_^(*i*)^]_1_ = 0, where *m* = −*M*,…, *M*. If *w*_*QS*_(*m*) = *q*_*m*_(*θ*_*d*_, *ϕ*_*d*_)(*a*_*d*2_* + *ja*_*d*1_*), where *a*_*d*1_ = *a*_1_(*θ*_*d*_, *ϕ*_*d*_, *α*_*d*_, *β*_*d*_) and *a*_*d*2_ = *a*_2_(*θ*_*d*_, *ϕ*_*d*_, *α*_*d*_, *β*_*d*_), this constraint is satisfied. Thus, we can obtain

(26) $$\begin{array}{c} w_{QS}=\mathrm{diag}\left(q_{d}\right)\left(a_{d2}^{*}+ja_{d1}^{*}\right), \end{array}$$

where diag⁡(**q**_*d*_) = diag⁡{*q*_−*M*_(*θ*_*d*_, *ϕ*_*d*_),…, *q*_*M*_(*θ*_*d*_, *ϕ*_*d*_)}.

Inserting **G** = **w**_*QS*_ **w**_*MV*_ into (24), *y*_*g*_(*n*) can be rewritten as

(27) $$\begin{array}{c} y_{g}\left(n\right)=w_{MV}^{H}{\left[w_{QS}^{⊲}v_{u}^{\left(i\right)}\right]}_{1}s_{u}\left(n\right)+w_{MV}^{H}{\left[w_{QS}^{⊲}N^{\left(i\right)}\left(n\right)\right]}_{1}. \end{array}$$

Then, **w**_*MV*_ can be derived by solving the following constrained optimization problem:

(28) J(wMV)=min⁡{wMVHRQSwMV},subject  to  wMVHV~u=W−HV−u,

where **R**_*QS*_ = *E*{[**w**_*QS*_^⊲^ **x** ^(*i*)^(*n*)]_1_[**w**_*QS*_^⊲^ **x** ^(*i*)^(*n*)]_1_^*H*^} is the covariance matrix and ${\tilde{V}}_{u}={\left[w_{QS}^{⊲}v_{u}^{\left(i\right)}\right]}_{1}$. The solution of this constrained optimization problem is obtained by using Lagrange multipliers; that is,

(29) $$\begin{array}{c} w_{MV}=\frac{R_{QS}^{-1}{\tilde{V}}_{u}}{{\tilde{V}}_{u}^{H}R_{QS}^{-1}{\tilde{V}}_{u}}{\overset{-}{V}}_{u}^{H}\overset{-}{W}. \end{array}$$

If the desired signal and interference are uncorrelated with the additive noise, **w**_*MV*_ can be written in the simple form (the proof is in Appendix B)

(30) $$\begin{array}{c} w_{MV}=\frac{g_{1}}{\kappa }{\tilde{V}}_{u}, \end{array}$$

where

(31) κ=V~uHV~u=2M(|ad2|2|au1|2+|ad1|2|au2|2) −2R(ad1ad2∗au2au1∗(qu2)Hqd2),g1=(W−HV−u)H=ξu−1[Pu⊲Pd]1quHqdμ,

where *R*(·) denotes the real part of a complex number. *μ* is given by (20).

#### 3.2.2. Scheme  2, That Is, LCMV

In this scheme, we employ the LCMV beamformer as the second-stage beamformer. Since the* Cayley-Dickson* representation of **G**, **v**_*d*_^(*i*)^, **v**_*u*_^(*i*)^, and **N** ^(*i*)^(*n*) are, respectively, **G** = **G**_1_ + *j* **G**_2_, **v**_*d*_^(*i*)^ = **v**_*d*1_ − *j* **v**_*d*2_, **v**_*u*_^(*i*)^ = **v**_*u*1_ − *j* **v**_*u*2_, and **N** ^(*i*)^(*n*) = **N**_1_(*n*) − *j* **N**_2_(*n*), we have

(32) $$\begin{array}{c} {\left[G^{⊲}v_{d}^{\left(i\right)}\right]}_{1}=G_{1}^{H}v_{d1}-G_{2}^{H}v_{d2}={\overset{-}{G}}^{H}{\overset{-}{V}}_{d}^{\left(i\right)}, \end{array}$$

(33) $$\begin{array}{c} {\left[G^{⊲}v_{u}^{\left(i\right)}\right]}_{1}=G_{1}^{H}v_{u1}-G_{2}^{H}v_{u2}={\overset{-}{G}}^{H}{\overset{-}{V}}_{u}^{\left(i\right)}, \end{array}$$

(34) $$\begin{array}{c} {\left[G^{⊲}N^{\left(i\right)}(n)\right]}_{1}=G_{1}^{H}N_{1}\left(n\right)-G_{2}^{H}N_{2}\left(n\right)={\overset{-}{G}}^{H}{\overset{-}{N}}^{\left(i\right)}\left(n\right), \end{array}$$

where

(35) $$\begin{array}{c} \overset{-}{G}=\left[\begin{array}{c} G_{1}\\ G_{2} \end{array}\right],\;\;{\overset{-}{V}}_{d}^{\left(i\right)}=\left[\begin{array}{c} v_{d1}\\ -v_{d2} \end{array}\right],\\ {\overset{-}{V}}_{u}^{\left(i\right)}=\left[\begin{array}{c} v_{u1}\\ -v_{u2} \end{array}\right],\;\;{\overset{-}{N}}^{\left(i\right)}\left(n\right)=\left[\begin{array}{c} N_{1}\left(n\right)\\ -N_{2}\left(n\right) \end{array}\right]. \end{array}$$

Thus, (24) can be rewritten as

(36) $$\begin{array}{c} y_{g}\left(n\right)={\overset{-}{G}}^{H}{\overset{-}{V}}_{d}^{\left(i\right)}s_{d}\left(n\right)+{\overset{-}{G}}^{H}{\overset{-}{V}}_{u}^{\left(i\right)}s_{u}\left(n\right)+{\overset{-}{G}}^{H}{\overset{-}{N}}^{\left(i\right)}\left(n\right). \end{array}$$

Then, $\overset{-}{G}$ can be derived by solving the following constrained optimization problem:

(37) $$\begin{array}{c} J\left(\overset{-}{G}\right)=\min\left\{{\overset{-}{G}}^{H}R_{inG}\overset{-}{G}\right\},\;\text{subject}\;\;\text{to}\;\;{\overset{-}{G}}^{H}C=g^{H}, \end{array}$$

where

(38) $$\begin{array}{c} R_{inG}=\left[\begin{array}{cc} E\left\{{\left[x_{in}^{i}\left(n\right)\right]}_{1}{\left[x_{in}^{i}\left(n\right)\right]}_{1}^{H}\right\} & E\left\{{\left[x_{in}^{i}\left(n\right)\right]}_{1}{\left[x_{in}^{i}\left(n\right)\right]}_{2}^{H}\right\}\\ E\left\{{\left[x_{in}^{i}\left(n\right)\right]}_{2}{\left[x_{in}^{i}\left(n\right)\right]}_{1}^{H}\right\} & E\left\{{\left[x_{in}^{i}\left(n\right)\right]}_{2}{\left[x_{in}^{i}\left(n\right)\right]}_{2}^{H}\right\} \end{array}\right] \end{array}$$

is the covariance matrix and **x**_*in*_^*i*^(*n*) = **v**_*u*_^*i*^ *s*_*u*_(*n*) + **N** ^*i*^(*n*) is the quaternion involution of **x**_*in*_(*n*). $C=[{\overset{-}{V}}_{u}^{\left(i\right)},{\overset{-}{V}}_{d}^{\left(i\right)}]$ and **g** ^*H*^ = [*g*_1_^*H*^, 0], where *g*_1_ is given by (31). The solution of (37) is given by [30]

(39) $$\begin{array}{c} \overset{-}{G}=R_{inG}^{-1}C{\left(C^{H}R_{inG}^{-1}C\right)}^{-1}g. \end{array}$$

If the desired signal and interference are uncorrelated with the additive noise, $\overset{-}{G}$ can be written in the simple form (the proof is in Appendix C)

(40) $$\begin{array}{c} \overset{-}{G}=\frac{g_{1}}{\nu }\left(2M{\left|P_{d}\right|}^{2}{\overset{-}{V}}_{u}^{\left(i\right)}-{\left[P_{d}^{⊲}P_{u}\right]}_{1}q_{d}^{H}q_{u}{\overset{-}{V}}_{d}^{\left(i\right)}\right), \end{array}$$

where

(41) ν=(2M)2|Pd|2|Pu|2−|[Pu⊲Pd]1|2|quHqd|2=μ−ξu−12M|Pd|2,

where *μ* is given by (20). Moreover, the quaternion-valued optimal weight vector **G** may be given by

(42) $$\begin{array}{c} G=J_{1}\overset{-}{G}+jJ_{2}\overset{-}{G}, \end{array}$$

where **J**_1_ = [**I**_2*M*×2*M*_, 0_2*M*×2*M*_] and **J**_2_ = [0_2*M*×2*M*_, **I**_2*M*×2*M*_] are two selection matrices.

By using the optimal weight vector **G**, the complex output of second-stage beamformer can be given by

(43) $$\begin{array}{c} y_{g}\left(n\right)={\left[W^{⊲}v_{u}\right]}_{1}s_{u}\left(n\right)+{\left[G^{⊲}N^{\left(i\right)}\left(n\right)\right]}_{1}. \end{array}$$

Thus, the complex output of QSWL GSC may be rewritten as

(44) yGSC(n)=yw(n)−yg(n)=sd(n)+[W⊲N(n)]1−[G⊲N(i)(n)]1.

From the above equation, we see that the interference component is completely canceled in the output *y*_GSC_(*n*).

### 3.3. The Performance Analysis

Since the QSWL GSC can totally remove the interference, its output signal-to-interference ratio (SIR) tends to infinite. Thus, we focus our attention on the output signal-to-noise ratio (SNR) and array's gain. Let *p*_*n*_ = *E*{|[**W** ^⊲^ **N**(*n*)]_1_ − [**G** ^⊲^ **N** ^(*i*)^(*n*)]_1_|^2^} be the power of output noise. From (13) and (34), we have

(45) [W⊲N(n)]1−[G⊲N(i)(n)]1 =W1HN1(n)+W2HN2(n)  −G1HN1(n)+G2HN2(n) =(W1H−G1H)N1(n)+(W2H+G2H)N2(n).

Then, *p*_*n*_ can be written as

(46) pn=σn2(W1H−G1H)(W1−G1) +σn2(W2H+G2H)(W2+G2)=σn2(W−HW−+G−HG−− W1HG1+W2HG2   −G1HW1+G2HW2).

When the combined QPMC and MVDR are adopted in the second-stage beamformer, *p*_*n*_ can be written in the simple form (the proof is in Appendix D)

(47) $$\begin{array}{c} p_{n}=\frac{\sigma_{n}^{2}}{\mu^{2}}\left(2M{\left|P_{d}\right|}^{2}\varepsilon^{2}+{\left|{\left[P_{u}^{⊲}P_{d}\right]}_{1}\right|}^{2}{\left|q_{u}^{H}q_{d}\right|}^{2}\gamma_{q}\right), \end{array}$$

where

(48) $$\begin{array}{c} \gamma_{q}=\frac{\xi_{u}^{-2}{\left|P_{d}\right|}^{2}}{\kappa }-2M{\left|P_{u}\right|}^{2}, \end{array}$$

where *κ* is given by (31). From (44), the expression of output SNR and array's gain *A*_*q*_ may be written as

(49) $$\begin{array}{c} {\text{SNR}}_{o}=\xi_{d}\frac{{\left(2M{\left|P_{d}\right|}^{2}\varepsilon -{\left|{\left[P_{u}^{⊲}P_{d}\right]}_{1}\right|}^{2}{\left|q_{u}^{H}q_{d}\right|}^{2}\right)}^{2}}{2M{\left|P_{d}\right|}^{2}\varepsilon^{2}+{\left|{\left[P_{u}^{⊲}P_{d}\right]}_{1}\right|}^{2}{\left|q_{u}^{H}q_{d}\right|}^{2}\gamma_{q}},\\ A_{q}=\frac{{\left(2M{\left|P_{d}\right|}^{2}\varepsilon -{\left|{\left[P_{u}^{⊲}P_{d}\right]}_{1}\right|}^{2}{\left|q_{u}^{H}q_{d}\right|}^{2}\right)}^{2}}{2M{\left|P_{d}\right|}^{2}\varepsilon^{2}+{\left|{\left[P_{u}^{⊲}P_{d}\right]}_{1}\right|}^{2}{\left|q_{u}^{H}q_{d}\right|}^{2}\gamma_{q}}, \end{array}$$

where *ξ*_*d*_ denotes the input signal-to-noise ratio (SNR) and *ε* is given by (20).

When the LCMV is adopted in the second-stage beamformer, *p*_*n*_ can be written in the simple form (the proof is in Appendix E)

(50) $$\begin{array}{c} p_{n}=\frac{\sigma_{n}^{2}}{\mu^{2}}\left(2M{\left|P_{d}\right|}^{2}\varepsilon^{2}+{\left|{\left[P_{u}^{⊲}P_{d}\right]}_{1}\right|}^{2}{\left|q_{u}^{H}q_{d}\right|}^{2}\gamma_{l}\right), \end{array}$$

where

(51) $$\begin{array}{c} \gamma_{l}=\frac{2M\xi_{u}^{-2}{\left|P_{d}\right|}^{2}}{\nu }-2M{\left|P_{u}\right|}^{2}, \end{array}$$

where *ν* is given by (41). Then, the expression of output SNR and array's gain *A*_*l*_ may be written as

(52) $$\begin{array}{c} {\text{SNR}}_{o}=\xi_{d}\frac{{\left(2M{\left|P_{d}\right|}^{2}\varepsilon -{\left|{\left[P_{u}^{⊲}P_{d}\right]}_{1}\right|}^{2}{\left|q_{u}^{H}q_{d}\right|}^{2}\right)}^{2}}{2M{\left|P_{d}\right|}^{2}\varepsilon^{2}+{\left|{\left[P_{u}^{⊲}P_{d}\right]}_{1}\right|}^{2}{\left|q_{u}^{H}q_{d}\right|}^{2}\gamma_{l}},\\ A_{l}=\frac{{\left(2M{\left|P_{d}\right|}^{2}\varepsilon -{\left|{\left[P_{u}^{⊲}P_{d}\right]}_{1}\right|}^{2}{\left|q_{u}^{H}q_{d}\right|}^{2}\right)}^{2}}{2M{\left|P_{d}\right|}^{2}\varepsilon^{2}+{\left|{\left[P_{u}^{⊲}P_{d}\right]}_{1}\right|}^{2}{\left|q_{u}^{H}q_{d}\right|}^{2}\gamma_{l}}. \end{array}$$

From (49) and (52), we can see that the output SNR and array's gain depend on not only separation between the DOA's of the desired signal and interference (i.e., |**q**_*u*_^*H*^ **q**_*d*_|), but also difference between the polarization of the desired signal and interference (i.e., |[*P*_*u*_^⊲^ *P*_*d*_]_1_|). The dependency of them on |**q**_*u*_^*H*^ **q**_*d*_| and |[*P*_*u*_^⊲^ *P*_*d*_]_1_| is shown in the following consequences.

(1) When |**q**_*u*_^*H*^ **q**_*d*_ | = 0, the separation between the DOAs of the desired signal and interference reaches to the maximum. In this case, *A*_*q*_ = *A*_*l*_ = 2*M* | *P*_*d*_|^2^. Further, |**q**_*u*_^*H*^ **q**_*d*_| increases with the decrease of the DOA's separation. Thus, the array's gain of *A*_*q*_ and *A*_*l*_ will reduce if |*P*_*d*_|^2^ is a constant. When **q**_*u*_ = **q**_*d*_, |**q**_*u*_^*H*^ **q**_*d*_ | = 2*M*. This implies that there is no separation between the DOAs of the desired signal and interference. In this case, the array's gain is given by
  (53) $$\begin{array}{c} A_{q}=A_{l}=\frac{2M{\left({\left|P_{d}\right|}^{2}\varepsilon -2M{\left|{\left[P_{u}^{⊲}P_{d}\right]}_{1}\right|}^{2}\right)}^{2}}{{\left|P_{d}\right|}^{2}\varepsilon^{2}+2M{\left|{\left[P_{u}^{⊲}P_{d}\right]}_{1}\right|}^{2}\gamma }, \end{array}$$
  where
  (54) $$\begin{array}{c} \gamma =\frac{\xi_{u}^{-2}{\left|P_{d}\right|}^{2}}{2M\left({\left|P_{d}\right|}^{2}{\left|P_{u}\right|}^{2}-{\left|{\left[P_{u}^{⊲}P_{d}\right]}_{1}\right|}^{2}\right)}-2M{\left|P_{u}\right|}^{2}. \end{array}$$
  Further, *P*_*u*_ = *P*_*d*_ if *α*_*d*_ = *α*_*u*_ and *β*_*d*_ = *β*_*u*_. Thus, the array's gain *A*_*q*_ = *A*_*l*_ = 0 because *γ* = *∞*. This implies that the QSWL GSC fails.
(2) When |[*P*_*u*_^⊲^ *P*_*d*_]_1_ | = 0, *A*_*q*_ = *A*_*l*_ = 2*M* | *P*_*d*_|^2^. In the cases that *θ*_*d*_ = *θ*_*u*_ ≠ 0 and *ϕ*_*d*_ = *ϕ*_*u*_ ≠ 0 (i.e., **q**_*u*_ = **q**_*d*_), we have |[*P*_*u*_^⊲^ *P*_*d*_]_1_ | = (sin^2^ *θ*_*d*_cos⁡^2^ *ϕ*_*d*_ + sin^2^ *ϕ*_*d*_)cos⁡ (*α*_*u*_ − *α*_*d*_)cos⁡ (*β*_*u*_ − *β*_*d*_). If *α*_*u*_ − *α*_*d*_ = ±*π*/2 or *β*_*u*_ − *β*_*d*_ = ±*π*/2, then |[*P*_*u*_^⊲^ *P*_*d*_]_1_ | = 0. This implies that, even though there is no separation between the DOAs of the desired signal and interference, the array's gain can also reach 2*M* | *P*_*d*_|^2^ by using the orthogonality between the polarization of the desired signal and interference. Further, the array's gain decreases with the increase of |[*P*_*u*_^⊲^ *P*_*d*_]_1_| if |*P*_*d*_|^2^ is a constant. When *P*_*u*_ = *P*_*d*_, |[*P*_*u*_^⊲^ *P*_*d*_]_1_ | = |*P*_*d*_|^2^. This implies that there is no difference between the polarization of the desired signal and interference. But, the array's gain is not equal to zero if **q**_*u*_ ≠ **q**_*d*_.

In addition, the output SNR and array's gain depend also on the input INR *ξ*_*u*_, the array's element number 2*M*, the interference response's power |*P*_*u*_|^2^, and the desired signal response's power |*P*_*d*_|^2^.

## 4. Monte Carlo Simulations

In this section, we investigate the performance of the proposed QSWL GSC (i.e., QSWL-QPMC-MVDR and QSWL-LCMV). In simulations, we consider a two-component vector-sensor array depicted in Figure 1, where each two-component vector sensor consists of one electric dipole and one magnetic loop coaligned along the *x*-axis. For each two-component vector sensor, the responses on the first and second components are given by

(55) [a1(θ,ϕ,α,β)a2(θ,ϕ,α,β)]=[−sinϕ−sinθcos⁡ϕ−sinθcos⁡ϕsinϕ]  ×[cos⁡αsinα−sinαcos⁡α][cos⁡βisinβ],

where *θ* ∈ (−*π*/2, *π*/2]; *ϕ* ∈ (−*π*, *π*]; *α* ∈ (−*π*/2, *π*/2]; and *β* ∈ [−*π*/4, *π*/4]. The spatial phase factor vector **q**(*θ*, *ϕ*) = [*e* ^−*i*(*Mπ*/2)sin*θ*cos⁡*ϕ*^,…, *e* ^*i*(*Mπ*/2)sin*θ*cos⁡*ϕ*^]^*T*^. To prevent performance degradation in the presence of DOA mismatch and/or array perturbations, we use the three main-lobe constraints [30], that is, $C=[{\overset{-}{V}}_{d}(\theta_{d}),\;{\overset{-}{V}}_{d}(\theta_{d}-\theta_{c}),\;{\overset{-}{V}}_{d}(\theta_{d}+\theta_{c})]$ and **g** = [1,1, 1]^*H*^, in all experiments. It is assumed that *ξ*_*d*_ = 10 and *ξ*_*u*_ = 1. To compare the performance, the complex “long vector" LCMV (CLCMV) beamformer [30–32] is also included in simulation results.

### 4.1. Performance for One Type of Interference

**Figure 3:**
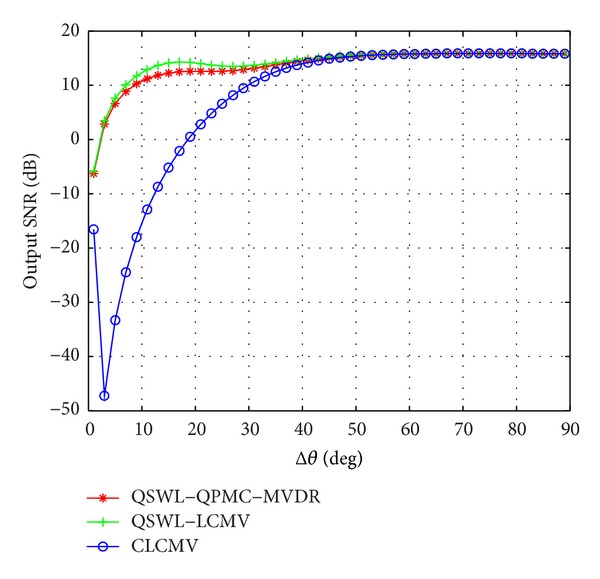
Effect of the angular separation Δ*θ* between the interference and the desired signal.

In the presence of a single interference, we illustrate the performance of the proposed QSWL GSC by employing two experiments. We assume that *M* = 6, *ϕ*_*u*_ = *ϕ*_*d*_ = 60°, *α*_*u*_ = *α*_*d*_ = 30°, *β*_*u*_ = *β*_*d*_ = 30°, and *θ*_*c*_ = 1°. In the first experiment, we investigate the effect of the angular separation Δ*θ* between the desired signal and the interference, where Δ*θ* = *θ*_*u*_ − *θ*_*d*_.  Figure 3 displays the output SINR as a function of Δ*θ*, where *θ*_*u*_ = Δ*θ*, *θ*_*d*_ = 0°. From Figure 3, it is seen that the output SINR of the proposed QSWL GSC is larger than that of the CLCMV in small angular separation (i.e., Δ*θ* < 40°). Compared with the QSWL-QPMC-MVDR, the QSWL-LCMV has a little increase of the output SINR. This implies that the QSWL GSC has the better performance in small angular separation.

**Figure 4:**
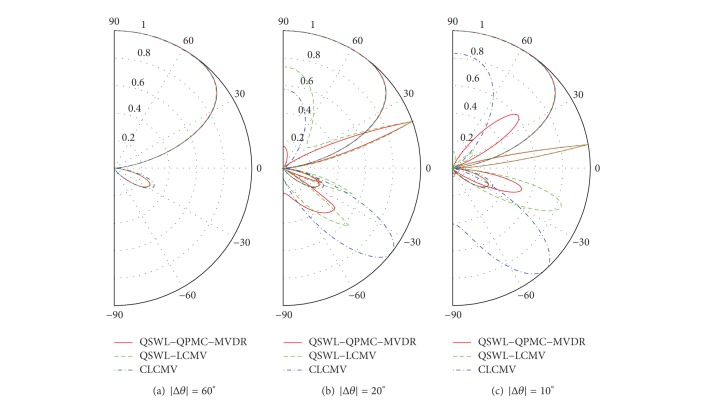
The power patterns at *θ*_*u*_ = 0° and *θ*_*d*_ = |Δ*θ*|.

In the second experiment, we assume that the covariance matrix **R**_*x*_, instead of **R**_*in*_, is available.  Figure 4 displays the power patterns for three values of |Δ*θ*|: 60°, 20°, and 10°, where *θ*_*d*_ = |Δ*θ*|, *θ*_*u*_ = 0°. From Figure 4, it is seen that the power is almost zero towards the interference's DOA (located at 0°) in all cases. When |Δ*θ*| decreases, the main lobe of the QSWL GSC points almost to the source location, but the main lobe of the CLCMV is away from the source location. This implies that the QSWL GSC outperforms obviously CLCMV as the desired signal moves towards the interference. In addition, the side lobes are amplified with a decrease of |Δ*θ*|. These side lobes lead the beamformer to capture the white noise which spans the whole space, so that the performance of beamformer degrades.

### 4.2. Performance for Two Types of Interference

**Figure 5:**
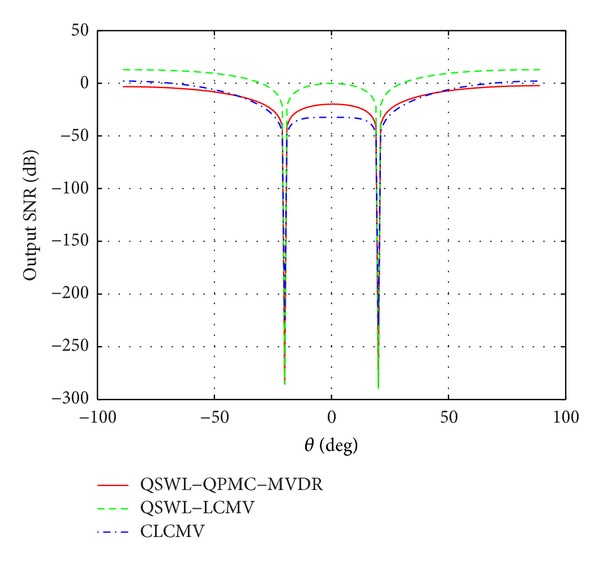
Effect of the angular mismatch between the distortionless constraint direction and the real arrival direction of the desired signal at *M* = 3.

**Figure 6:**
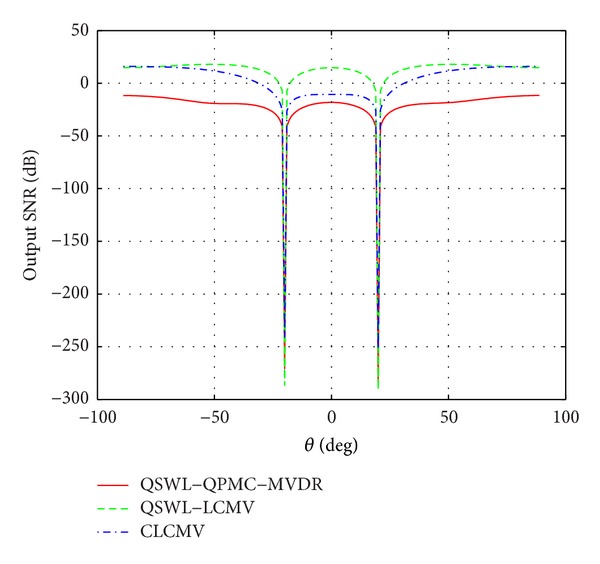
Effect of the angular mismatch between the distortionless constraint direction and the real direction of the desired signal at *M* = 6.

In the presence of several types of interference, the performance analysis of the proposed QSWL GSC is not so easy in theory. Thus, we employ two experiments to illustrate the performance of the proposed QSWL GSC. We assume that there are two types of interference with identical input INR, *ϕ*_*d*_ = *ϕ*_*u*1_ = *ϕ*_*u*2_ = 60°, *α*_*d*_ = *α*_*u*1_ = *α*_*u*2_ = 30°, *β*_*d*_ = *β*_*u*1_ = *β*_*u*2_ = 30°, and *θ*_*c*_ = 3°. The covariance matrix **R**_*x*_ is used. In the first experiment, we investigate the effect of DOA mismatch, where *θ* is used to denote the real presumed DOA of the desired signal. Figures 5 and 6 display, respectively, the output SINR as a function of *θ* at *M* = 3 and *M* = 6, where *θ*_*d*_ = 0°, *θ*_*u*1_ = −20°, and *θ*_*u*2_ = 20°. From Figures 5 and 6, it is seen that the QSWL-LCMV beamformer outperforms obviously both the QSWL-QPMC-MVDR and the CLCMV. In the case of no DOA mismatch and *M* = 6, the output SINR of the QSWL-LCMV is 25 dB more than that of the CLCMV, but the output SINR of the CLCMV is larger than that of the QSWL-QPMC-MVDR. When the array's element number reduces, the performance of the CLCMV degrades. At *M* = 3, the output SINR of the QSWL-QPMC-MVDR are 12 dB more than that of the CLCMV. The cause of this behavior is that the QSWL GSC has larger degrees of freedom than the CLCMV. Thus, the QSWL GSC has the better performance when many constraints are simultaneously imposed and/or the array's element number is reduced. In addition, when the DOA mismatch is little, such as *θ* ≤ 10°, the output SINR of three beamformers do not obviously degrade.

**Figure 7:**
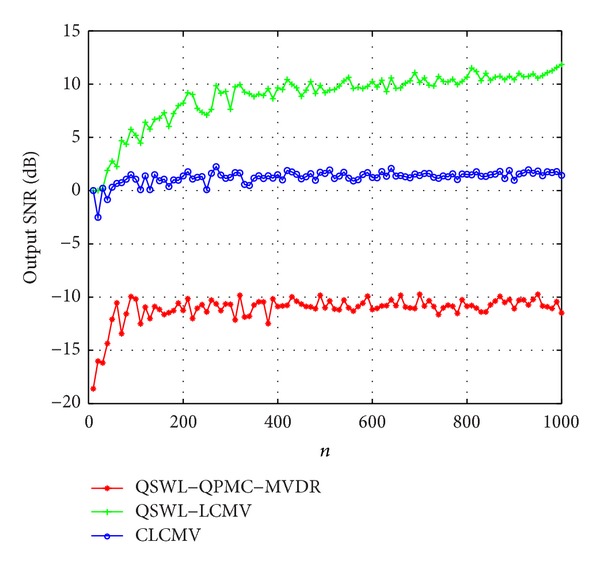
Effect of the number of snapshots at *M* = 6.

In the second experiment, we investigate the effect of snapshots *n*, where the covariance matrix **R**_*x*_ is replaced by the sample covariance matrix ${\hat{R}}_{x}$.  Figure 7 displays the output SINR as a function of *n* at *M* = 6, where *θ*_*d*_ = 0°, *θ*_*u*1_ = −30°, and *θ*_*u*2_ = 30°. From Figure 7, it is seen that the QSWL GSC has slower convergence than the CLCMV. But the output SINR of the QSWL-LCMV are obviously more than that of the CLCMV.

## 5. Conclusion

In this paper, we propose a quaternion* semiwidely* linear beamformer and its useful implementation based on a quaternion model of linear symmetric array with 2*M* two-component EM vector sensors. Since the QSWL GSC consists of two-stage beamformers, it has more information than the complex “long vector" beamformer. The increase of information results in the improvement of the beamformer's performance. By designing the weight vectors of two-stage beamformers, the interference is completely canceled in the output of QSWL GSC and the desired signal is not distorted. Simulation results reveal that the proposed QSWL GSC has the better performance in small angular separation and the robustness to DOA mismatch.

## Endnotes

1. According to the property of quaternion shown in [24], we have *jc* = *c***j* for any complex number *c*. Thus, *jx*_−*m*2_(*t*) = *q*_*m*_(*θ*, *ϕ*)*ja*_2_(*θ*, *ϕ*, *α*, *β*)*s*(*t*). Since the spatial phase factor *q*_*m*_(*θ*, *ϕ*) in *x*_*m*1_(*t*) is identical with that in *jx*_−*m*2_(*t*), we use *x*_−*m*2_(*t*) instead of *x*_*m*2_(*t*) to construct the quaternion-valued signal *x*_*m*_(*t*).

2. According to the definition in [11], **x** is *C* ^*i*^-proper if it can be represented by means of two jointly proper complex vectors in the plane spanned by {1, *i*}.

3. According to the definition in [11], the augmented covariance matrix of a *C* ^*i*^-proper quaternion vector **x** can be written as

(∗)

where $\overset{-}{x}=[x^{T},x^{(i)T},x^{(j)T},x^{(k)T}]$ is the augmented quaternion vector; $\tilde{x}=[x^{T},x^{(i)T}]$ is the semiaugmented quaternion vector; and $R_{\tilde{x},\tilde{x}}=E\{\tilde{x}{\tilde{x}}^{⊲}\}$ is the semiaugmented covariance matrix of quaternion vector **x**. In comparison with the semiaugmented covariance matrix $R_{\tilde{x},\tilde{x}}$, the augmented covariance matrix $R_{\overset{-}{x},\overset{-}{x}}$ has no more extra information. In other words, the* full widely* linear processing is equivalent to the* semiwidely* linear processing in handling the *C* ^*i*^-proper quaternion vector. We should not expect that the performance is improved by replacing* semiwidely* linear processing with* full widely* linear processing.

## Appendices

### A. **Derivation for (19)**

If the interference and additive noise are uncorrelated, we have

(A.1) $$\begin{array}{c} R_{in}=\sigma_{u}^{2}{\overset{-}{V}}_{u}{\overset{-}{V}}_{u}^{H}+\sigma_{n}^{2}I, \end{array}$$

where *σ*_*u*_^2^ and *σ*_*n*_^2^ are the covariance of interference and additive noise, respectively. By making use of the matrix inversion lemma (see Appendix in [30]), we find

(A.2) $$\begin{array}{c} R_{in}^{-1}=\frac{1}{\sigma_{n}^{2}}\left(I-\frac{{\overset{-}{V}}_{u}{\overset{-}{V}}_{u}^{H}}{\xi_{u}^{-1}+{\overset{-}{V}}_{u}^{H}{\overset{-}{V}}_{u}}\right), \end{array}$$

where *ξ*_*u*_ = *σ*_*u*_^2^/*σ*_*n*_^2^. Plugging (A.2) into (18), we obtain

(A.3) $$\begin{array}{c} \overset{-}{W}=\frac{{\overset{-}{V}}_{d}-\left({\overset{-}{V}}_{u}{\overset{-}{V}}_{u}^{H}{\overset{-}{V}}_{d}/\left(\xi_{u}^{-1}+{\overset{-}{V}}_{u}^{H}{\overset{-}{V}}_{u}\right)\right)}{{\overset{-}{V}}_{d}^{H}{\overset{-}{V}}_{d}-\left({\overset{-}{V}}_{d}^{H}{\overset{-}{V}}_{u}{\overset{-}{V}}_{u}^{H}{\overset{-}{V}}_{d}/\left(\xi_{u}^{-1}+{\overset{-}{V}}_{u}^{H}{\overset{-}{V}}_{u}\right)\right)}. \end{array}$$

Plugging ${\overset{-}{V}}_{k}^{H}{\overset{-}{V}}_{k}=2M|P_{k}{|}^{2}$ (*k* = *d*, *u*) and ${\overset{-}{V}}_{u}^{H}{\overset{-}{V}}_{d}={\left[P_{u}^{⊲}P_{d}\right]}_{1}q_{u}^{H}q_{d}$ into (A.3), we can obtain (19) after some manipulation.

### B. **Derivation for (30)**

If the interference and additive noise are uncorrelated, we have

(B.1) $$\begin{array}{c} R_{QS}=\sigma_{u}^{2}{\tilde{V}}_{u}{\tilde{V}}_{u}^{H}+\sigma_{n}^{2}{\left|P_{d}\right|}^{2}I. \end{array}$$

By making use of the matrix inversion lemma, we find

(B.2) $$\begin{array}{c} R_{QS}^{-1}=\frac{1}{\sigma_{n}^{2}{\left|P_{d}\right|}^{2}}\left(I-\frac{{\tilde{V}}_{u}{\tilde{V}}_{u}^{H}}{\xi_{u}^{-1}{\left|P_{d}\right|}^{2}+{\tilde{V}}_{u}^{H}{\tilde{V}}_{u}}\right). \end{array}$$

Plugging (B.2) into (29), we obtain

(B.3) $$\begin{array}{c} w_{MV}=\frac{{\tilde{V}}_{u}-\left({\tilde{V}}_{u}{\tilde{V}}_{u}^{H}{\tilde{V}}_{u}/\left(\xi_{u}^{-1}{\left|P_{d}\right|}^{2}+{\tilde{V}}_{u}^{H}{\tilde{V}}_{u}\right)\right)}{{\tilde{V}}_{u}^{H}{\tilde{V}}_{u}-\left({\tilde{V}}_{u}^{H}{\tilde{V}}_{u}{\tilde{V}}_{u}^{H}{\tilde{V}}_{u}/\left(\xi_{u}^{-1}{\left|P_{d}\right|}^{2}+{\tilde{V}}_{u}^{H}{\tilde{V}}_{u}\right)\right)}{\overset{-}{V}}_{u}^{H}\overset{-}{W}. \end{array}$$

Plugging ${\tilde{V}}_{u}^{H}{\tilde{V}}_{u}=\kappa$ and ${\overset{-}{V}}_{u}^{H}\overset{-}{W}=g_{1}$ into (B.3), we can obtain (30) after some manipulation.

### C. **Derivation for (40)**

Since $C=[{\overset{-}{V}}_{u}^{\left(i\right)},{\overset{-}{V}}_{d}^{\left(i\right)}]$, the inverse of 2 × 2 matrix **C** ^*H*^ **R**_*in**G*_^−1^ **C** is given by

(C.1) (CHRinG−1C)−1 =1τ[V−d(i)HRinG−1V−d(i)−V−u(i)HRinG−1V−d(i)−V−d(i)HRinG−1V−u(i)V−u(i)HRinG−1V−u(i)],

where

(C.2) τ=V−u(i)HRinG−1V−u(i)V−d(i)HRinG−1V−d(i) −V−u(i)HRinG−1V−d(i)V−d(i)HRinG−1V−u(i)

is the determinant of 2 × 2 matrix **C** ^*H*^ **R**_*in**G*_^−1^ **C**. Plugging (C.1) into (39) and after some manipulation, we obtain

(C.3) G−=g1τ(RinG−1V−u(i)V−d(i)HRinG−1V−d(i)) −g1τ(RinG−1V−d(i)V−d(i)HRinG−1V−u(i)).

By making use of the matrix inversion lemma, we find

(C.4) $$\begin{array}{c} R_{inG}^{-1}{\overset{-}{V}}_{u}^{\left(i\right)}=\frac{1}{\sigma_{n}^{2}}\frac{\xi_{u}^{-1}{\overset{-}{V}}_{u}^{\left(i\right)}}{\xi_{u}^{-1}+{\overset{-}{V}}_{u}^{(i)H}{\overset{-}{V}}_{u}^{\left(i\right)}},\\ R_{inG}^{-1}{\overset{-}{V}}_{d}^{\left(i\right)}=\frac{1}{\sigma_{n}^{2}}\left({\overset{-}{V}}_{d}^{\left(i\right)}-\frac{{\overset{-}{V}}_{u}^{\left(i\right)}{\overset{-}{V}}_{u}^{(i)H}{\overset{-}{V}}_{d}^{\left(i\right)}}{\xi_{u}^{-1}+{\overset{-}{V}}_{u}^{(i)H}{\overset{-}{V}}_{u}^{\left(i\right)}}\right). \end{array}$$

Plugging (C.4) into (C.3) and using the results ${\overset{-}{V}}_{k}^{(i)H}{\overset{-}{V}}_{k}^{\left(i\right)}=2M|P_{k}{|}^{2}$ (*k* = *d*, *u*); ${\overset{-}{V}}_{u}^{(i)H}{\overset{-}{V}}_{d}^{\left(i\right)}={\left[P_{u}^{⊲}P_{d}\right]}_{1}q_{u}^{H}q_{d}$; and ${\overset{-}{V}}_{d}^{(i)H}{\overset{-}{V}}_{u}^{\left(i\right)}={\left({\overset{-}{V}}_{u}^{(i)H}{\overset{-}{V}}_{d}^{\left(i\right)}\right)}^{H}={\left[P_{d}^{⊲}P_{u}\right]}_{1}q_{d}^{H}q_{u}$, we can obtain (40) after some manipulation.

### D. **Derivation for (47)**

Using (19), we can easily obtain

(D.1) W−HW−=2Mε2|Pd|2μ2 −(2ξu−1+2M|Pu|2)|[Pu⊲Pd]1|2|quHqd|2μ2.

Using the results **w**_*QS*_^⊲^ **w**_*QS*_ = |*P*_*d*_|^2^ and **w**_*MV*_^*H*^ **w**_*MV*_ = |*g*_1_|^2^/*κ*, we can easily obtain

(D.2) G−HG−=G⊲G=(wQSwMV)⊲(wQSwMV)=|Pd|2ξu−2|[Pu⊲Pd]1|2|quHqd|2μ2κ.

Using (19) and (26), we can easily obtain

(D.3) W1HG1=εVd1Hdiag⁡(qd)wMVad2∗μ −([Pu⊲Pd]1quHqd)HVu1Hdiag⁡(qd)wMVad2∗μ=εad1∗ad2∗μqdHdiag⁡(qd)wMV −([Pu⊲Pd]1quHqd)Hau1∗ad2∗μquHdiag⁡(qd)wMV,

(D.4) W2HG2=εVd2Hdiag⁡(qd∗)wMVad1∗μ −([Pu⊲Pd]1quHqd)HVu2Hdiag⁡(qd∗)wMVad1∗μ=εad1∗ad2∗μ(qdHdiag⁡(qd))∗wMV −([Pu⊲Pd]1quHqd)Hau2∗ad1∗μ(quHdiag⁡(qd))∗wMV.

Using **q**_*d*_^*H*^diag⁡(**q**_*d*_) = (**q**_*d*_^*H*^diag⁡(**q**_*d*_))* and **q**_*ud*_ = **q**_*u*_^*H*^diag⁡(**q**_*d*_), we can obtain

(D.5) W1HG1−W2HG2 =−([Pu⊲Pd]1quHqd)Hμ  ×(au1∗ad2∗qud−au2∗ad1∗qud∗)wMV.

Using (30) and (31), we can easily obtain (*a*_*u*1_**a*_*d*2_***q**_*ud*_ − *a*_*u*2_**a*_*d*1_***q**_*ud*_*)**w**_*MV*_ = *g*_1_. Thus, we have

(D.6) W1HG1−W2HG2=−([Pu⊲Pd]1quHqd)Hg1μ=−ξu−1|[Pu⊲Pd]1|2|quHqd|2μ2,G1HW1−G2HW2=(W1HG1−W2HG2)H=−ξu−1|[Pu⊲Pd]1|2|quHqd|2μ2.

Plugging (D.1), (D.2), and (D.6) into (46), we can obtain (47) after some manipulation.

### E. **Derivation for (50)**

Using the results ${\overset{-}{V}}_{k}^{(i)H}{\overset{-}{V}}_{k}^{\left(i\right)}=2M|P_{k}{|}^{2}$ (*k* = *d*, *u*); ${\overset{-}{V}}_{u}^{(i)H}{\overset{-}{V}}_{d}^{\left(i\right)}={\left[P_{u}^{⊲}P_{d}\right]}_{1}q_{u}^{H}q_{d}$; and ${\overset{-}{V}}_{d}^{(i)H}{\overset{-}{V}}_{u}^{\left(i\right)}={\left({\overset{-}{V}}_{u}^{(i)H}{\overset{-}{V}}_{d}^{\left(i\right)}\right)}^{H}$, we have

(E.1) 2M|Pd|2ν=(2M|Pd|2V−u(i)−[Pd⊲Pu]1qdHquV−d(i))H  ×(2M|Pd|2V−u(i)−[Pd⊲Pu]1qdHquV−d(i)).

Using (40), we can easily obtain

(E.2) G−HG−=2M|Pd|2|g1|2ν=2M|Pd|2ξu−2|[Pu⊲Pd]1|2|quHqd|2μ2ν.

Using (19) and (40), we can easily obtain

(E.3) W1HG1=g1ε2M|Pd|2Vd1HVu1μν −g1ε[Pd⊲Pu]1qdHquVd1HVd1μν −g1([Pu⊲Pd]1quHqd)H2M|Pd|2Vu1HVu1μν +g1([Pu⊲Pd]1quHqd)H[Pd⊲Pu]1qdHquVu1HVd1μν,

(E.4) W2HG2=−g1ε2M|Pd|2Vd2HVu2μν +g1ε[Pd⊲Pu]1qdHquVd2HVd2μν +g1([Pu⊲Pd]1quHqd)H2M|Pd|2Vu2HVu2μν −g1([Pu⊲Pd]1quHqd)H[Pd⊲Pu]1qdHquVu2HVd2μν.

Using the results **V**_*k*1_^*H*^ **V**_*k*1_ + **V**_*k*2_^*H*^ **V**_*k*2_ = 2*M* | *P*_*k*_|^2^ (*k* = *d*, *u*); **V**_*d*1_^*H*^ **V**_*u*1_ + **V**_*d*2_^*H*^ **V**_*u*2_ = [*P*_*d*_^⊲^ *P*_*u*_]_1_ **q**_*d*_^*H*^ **q**_*u*_; and **V**_*u*1_^*H*^ **V**_*d*1_ + **V**_*u*2_^*H*^ **V**_*d*2_ = [*P*_*u*_^⊲^ *P*_*d*_]_1_ **q**_*u*_^*H*^ **q**_*d*_, we have

(E.5) W1HG1−W2HG2=−ξu−1|[Pu⊲Pd]1|2|quHqd|2μ2,G1HW1−G2HW2=(W1HG1−W2HG2)H=−ξu−1|[Pu⊲Pd]1|2|quHqd|2μ2.

Plugging (D.1), (E.2), and (E.5) into (46), we can obtain (50) after some manipulation.
